# Decreased brain venous vasculature visibility on susceptibility-weighted imaging venography in patients with multiple sclerosis is related to chronic cerebrospinal venous insufficiency

**DOI:** 10.1186/1471-2377-11-128

**Published:** 2011-10-19

**Authors:** Robert Zivadinov, Guy U Poloni, Karen Marr, Claudiu V Schirda, Christopher R Magnano, Ellen Carl, Niels Bergsland, David Hojnacki, Cheryl Kennedy, Clive B Beggs, Michael G Dwyer, Bianca Weinstock-Guttman

**Affiliations:** 1 Buffalo Neuroimaging Analysis Center, University at Buffalo, Buffalo, NY, USA; 2 The Jacobs Neurological Institute, University at Buffalo, Buffalo, NY, USA; 3 Centre for Infection Control and Biophysics, University of Bradford, Bradford, UK

## Abstract

**Background:**

The potential pathogenesis between the presence and severity of chronic cerebrospinal venous insufficiency (CCSVI) and its relation to clinical and imaging outcomes in brain parenchyma of multiple sclerosis (MS) patients has not yet been elucidated. The aim of the study was to investigate the relationship between CCSVI, and altered brain parenchyma venous vasculature visibility (VVV) on susceptibility-weighted imaging (SWI) in patients with MS and in sex- and age-matched healthy controls (HC).

**Methods:**

59 MS patients, 41 relapsing-remitting and 18 secondary-progressive, and 33 HC were imaged on a 3T GE scanner using pre- and post-contrast SWI venography. The presence and severity of CCSVI was determined using extra-cranial and trans-cranial Doppler criteria. Apparent total venous volume (ATVV), venous intracranial fraction (VIF) and average distance-from-vein (DFV) were calculated for various vein mean diameter categories: < .3 mm, .3-.6 mm, .6-.9 mm and > .9 mm.

**Results:**

CCSVI criteria were fulfilled in 79.7% of MS patients and 18.2% of HC (p < .0001). Patients with MS showed decreased overall ATVV, ATVV of veins with a diameter < .3 mm, and increased DFV compared to HC (all p < .0001). Subjects diagnosed with CCSVI had significantly increased DFV (p < .0001), decreased overall ATVV and ATVV of veins with a diameter < .3 mm (p < .003) compared to subjects without CCSVI. The severity of CCSVI was significantly related to decreased VVV in MS (p < .0001) on pre- and post-contrast SWI, but not in HC.

**Conclusions:**

MS patients with higher number of venous stenoses, indicative of CCSVI severity, showed significantly decreased venous vasculature in the brain parenchyma. The pathogenesis of these findings has to be further investigated, but they suggest that reduced metabolism and morphological changes of venous vasculature may be taking place in patients with MS.

## Background

Multiple sclerosis (MS) is considered a chronic, autoimmune, inflammatory disease of the central nervous system (CNS) characterized by inflammation, demyelination, axonal loss, and neurodegeneration [1].

Thin, linear periventricular white matter (WM) lesions (Dawson's fingers) present in the initial stages of MS are often oriented around the long axis of central veins [2]. Histopathological studies have confirmed the close relationship between inflammatory MS lesions and venous cerebral microvasculature, suggesting that the primary inflammatory process in MS regionalizes around blood vessels, with acute lesions showing lymphocytic perivascular infiltration, hypercellularity, macrophage infiltration and intra-macrophage myelin debris [3].

Early MR venography (MRV) studies suggest that a substantial number of MS lesions are crossed by well defined central veins [4]. These findings have been confirmed and extended by recent ultra-high-field 7T MRI studies which showed that a majority of MS lesions are associated with centrally coursing veins [5-7]. Close examination revealed well defined central veins surrounded by subtle abnormalities in signal intensities in a strict perivenous fashion, with vascular wall involvement [7,8].

Susceptibility-weighted imaging (SWI) venography can directly image cerebral veins using deoxyhemoglobin as an intrinsic contrast agent and, therefore, allows direct and non-invasive assessment of venous blood oxygenation saturation and visualization of the venous structures [9]. A recent pre-contrast SWI venography study showed significantly reduced periventricular WM venous vasculature visibility (VVV) in relapsing-remitting (RR) MS patients as compared to healthy control (HC) subjects [10].

The hypothesis that MS might be related to venous pathology has been considered in the past [3,11-13]. Recently, a condition called chronic cerebrospinal venous insufficiency (CCSVI) has been proposed and reported with high frequency in MS [14,15]. CCSVI is described as a vascular condition characterized by anomalies of the main extra-cranial cerebrospinal venous routes that interfere with normal blood outflow of brain parenchyma in patients with MS [14-16]. The pathogenesis between the presence and severity of CCSVI and its relation to clinical and imaging outcomes in brain parenchyma of MS patients has not yet been elucidated. In two recent pilot studies, by using perfusion-weighted imaging (PWI) and cerebrospinal fluid (CSF) imaging, we showed that there are drainage problems in patients with MS associated with presence and severity of CCSVI [17,18].

Therefore, the aim of this study was to extend our preliminary results by investigating the relationship between the presence and severity of CCSVI and altered VVV in the brain parenchyma on pre- and post-contrast SWI venography in patients with MS and in HC.

## Methods

This study was part of the Combined Trans-cranial and Extra-cranial Venous Doppler (CTEVD) study [19] that investigated the prevalence of CCSVI in patients with MS, clinically isolated syndrome (CIS), other neurological diseases (OND), and healthy controls (HC), using specific proposed Doppler criteria [14].

Fifty-nine (59) consecutive MS patients [41 RR and 18 secondary-progressive (SP)], and 33 age- and sex-matched consecutive HC were enrolled in this sub-study. Inclusion criteria were: RR or secondary-progressive (SP) disease course for MS patients, [20] age 18 to 65 years, Expanded Disability Status Scale (EDSS) [21] 0 to 6.5, and undergoing MRI scan with SWI venography. Exclusion criteria were: presence of relapse and steroid treatment in the 30 days preceding study entry for all patients, pre-existing medical conditions known to be associated with brain pathology (e.g., cerebrovascular disease, positive history of alcohol abuse, etc.), contraindication for having a contrast agent injected for MRI examination, history of cerebral congenital vascular malformations, or pregnancy.

Participants underwent a clinical and MRI examination and trans-cranial and extra-cranial Doppler scans of the head and neck. Doppler personnel were blinded to the subjects' status [19]. In particular, subjects were specifically instructed not to reveal their disease status during the Doppler examination. OND and CIS patients were not part of this CTEVD SWI venography sub-study, but were used as part of the overall population to ensure blinding in the CTEVD study. The MRI evaluators were completely blinded to subject's disease, clinical and CCSVI status.

The study was approved by the local Institutional Review Board and informed consent was obtained from all subjects.

### Doppler evaluation

The Doppler exam was performed using Esaote-Biosound MyLabGOLD25 ultrasound machine equipped with 2.5 and 7.5-10 MHz transducers and motorized chair capable of tilting from 0° to 90°. All study examinations were performed by the same Doppler technologist. We examined Doppler parameters that detect 5 anomalous venous hemodynamic (VH) criteria affecting cerebrospinal venous return, as previously described [14]. These include: 1) reflux in the IJV and/or in the VV assessed in both sitting and supine postures, 2) reflux in the deep cerebral veins (DCV), 3) B-mode detection of annuli, webs, septa, flaps or stenosis in the IJV, 4) absence of an ECD signal in the IJV and/or in the VV, even after forced deep breaths, and 5) presence of a negative difference in the cross sectional area of the IJV. More specific information is provided elsewhere [19].

Each subject was assigned a total VH criteria score which was calculated by counting the number of criteria that the subject fulfilled. A subject was considered CCSVI-positive, if ≥2 VH criteria were fulfilled, as previously proposed [14]. We also calculated the venous hemodynamic insufficiency severity score (VHISS) [18]. The overall VHISS score is defined as a weighted sum of the scores contributed by each individual VH criterion. The formula for VHISS calculations was: VHISS = VHISS1 + VHISS2 + VHISS3 + VHISS4 + VHISS5. The VHISS score is an ordinal measure of the overall extent and number of VH flow pattern anomalies, with a higher value of VHISS indicating a greater severity of VH flow pattern anomalies. The minimum possible VHISS value is 0 and the maximum 16.

### MRI acquisition and analysis

All subjects were examined on a 3 Tesla GE Signa Excite HD 12.0 Twin Speed 8-channel head coil scanner (General Electric, Milwaukee, WI).

SWI data was collected in all patients using a 3-Dimensional (3D) flow-compensated GRE (Gradient Recalled Echo) sequence with 64 locs/slab, 2 mm thick, a 512 × 192 matrix, FOV = 25.6 cm × 19.2 cm (512 × 256 matrix with in plane phase FOV = 0.75), for an in-plane resolution of 0.5 mm × 1 mm. Other acquisition parameters were: flip angle FA = 12, echo and repetition times (TE and TR) TE/TR = 22/40 ms, for an acquisition time AT = 8:46 min:sec. k-space data, as saved by the scanner (p-files) was saved and transferred to an offline Linux workstation for post processing using in-house developed software written in Matlab (MathWorks Inc.). Forty-eight of the 59 (81.4%) MS patients and 7 of 33 (21.1%) HC enrolled in the study obtained a second SWI scan after a single dose intravenous bolus of 0.1 mMol/Kg Gadolinium (Gd)-DTPA 10 min after injection. In order to test reproducibility and obtain preliminary data on our pre- and post-contrast experiment in HC, it was originally decided that 20-25% of the HC cohort be included in the contrast study. Additional MRI sequences acquired included 2D multi-planar dual fast spin-echo (FSE) proton density (PD) and T2-weighted image (WI), Fluid-Attenuated Inversion-Recovery (FLAIR), 3D high resolution (HIRES) T1-WI using a fast spoiled gradient echo (FSPGR) with magnetization-prepared inversion recovery (IR) pulse and spin echo (SE) T1-WI both with and without a single dose intravenous bolus of 0.1 mMol/Kg Gd-DTPA 5 min after injection (in 48 MS patients). The relevant parameters were as follows: for dual FSE PD/T2, echo and repetition times (TE and TR) TE1/TE2/TR = 9/98/5300 ms, flip angle (FA) = 90°, echo train length ETL = 14; for FLAIR, TE/TI/TR = 120/2100/8500 ms (inversion time, TI), FA = 90°, ETL = 24; for SE T1-WI, TE/TR = 16/600 ms, FA = 90; for 3D HIRES T1-WI, TE/TI/TR = 2.8/900/5.9 ms, FA = 10°. The 3D HIRES T1-WI scan was used for brain volume segmentation.

### SWI reconstruction

The k-space data, as saved by the scanner, include a preliminary zero filling in the z-direction. This is a standard feature of the sequence as implemented by the manufacturer and is not modifiable at the level of the graphical interface of the scanner. Data for each channel was reconstructed by zero-filling each slab location (slice) to 768 × 576, to produce images with an interpolated resolution of 0.33 mm × 0.33 mm. To ensure proper composition of multi-channel data for the magnitude and phase images, a channel re-centering and normalization process was employed, as previously described [22,23]. Following channel recombination, the magnitude image was processed using the non-parametric non-uniform intensity normalization program N3 [23,24]. Phase images were high-pass filtered using a 192 × 144 Hanning window for vein analysis, as suggested by Haacke et al. [25] The high-pass filtered phase image was used to generate a phase mask, as previously described [23,25]. SWI images were obtained by multiplying the phase mask four times onto the inhomogeneity-corrected magnitude image. SWI venograms were generated by performing a minimum Intensity Projection (mIP) over 5 2-mm thick sections and moving the mIP stack by one slice at a time.

### Vein extraction

**Figure 1:**
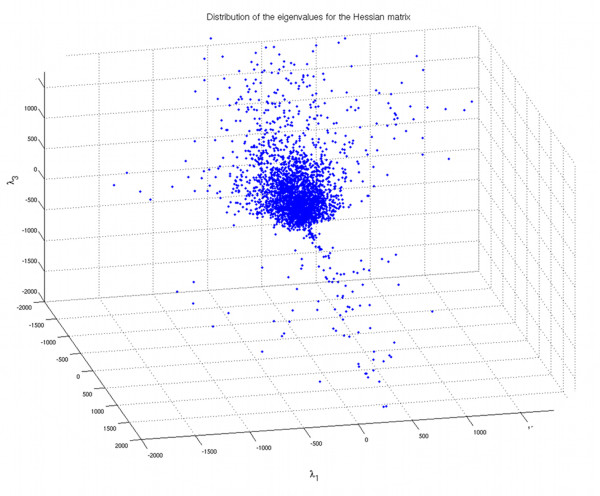
**Three-dimensional scatter plot of the eigenvalues of the Hessian matrix**. The voxels belonging to linear structures are those for which |λ_2_| and |λ_3_| are negative while |λ_1_| is close to zero, resulting in the cluster in the bottom part of the plot.

Quantification of SWI venous blood voxels was performed by extracting the venous structures of the brain from pre- and post-contrast SWI data using an in-house-developed segmentation method. The main component of the method is the vesselness filter described by Sato et al., [26] based on the second order information from the SWI mIP, represented by the Hessian matrix (the 3 × 3 matrix generated by the second derivatives of the image intensities in three spatial directions). By analyzing the eigenvalues (λ_j_) of the Hessian, a likeliness function was defined to distinguish tubular structures (vessels) from non-tubular structures (e.g., background noise, brain parenchyma, cerebrospinal fluid). The Hessian is calculated at a particular spatial scale that is varied to detect vessels of different diameters. The eigenvalues (λ_j_) of the Hessian, sorted by increasing magnitude (|λ_1_| < |λ_2_| < |λ_3_|), describe the local second order structure in an image; for instance, if λ_2 _and λ_3 _have a high value and λ_1 _is low, it means that the image intensity is constant or almost constant in a single direction only and changing rapidly in perpendicular directions, indicative of a tubular-like structure. The likeliness function was constructed to enhance dark tubular structures based on the eigenvalues and three tuning parameters: (a) defining the scale (σ), varied to detect vessels of different diameter, (b) defining the "level of vesselness" (α_1_), quantitatively specifying the qualitative relation |λ_1_| < < |λ_2_| & |λ_3_| characterizing tubular structures (Figure 1), and (c) restoring vessel continuity in fragmented parts (α_2_), where a higher noise level might result in fragmenting the curvilinear structures (Equation 1).

(1) $$f\left(\lambda_{1},\lambda_{2}\right)=\left\{\begin{array}{c} \exp\left(-\frac{\lambda_{1}^{2}}{2{\left(\alpha_{1}\lambda_{c}\right)}^{2}}\right)\lambda_{1}\leq 0,\lambda_{c}\neq 0\\ \exp\left(-\frac{\lambda_{1}^{2}}{2{\left(\alpha_{2}\lambda_{c}\right)}^{2}}\right)\lambda_{1}>0,\lambda_{c}\neq 0 \end{array}\right)$$

The values chosen for the aforementioned parameters were driven by those suggested by Sato et al., [26] as they are tuned to the size of the structures of interest and the image voxel size: 0.2 < σ < 2, α_1 _= 0.5, α_2 _= 2. One of the primary results of the paper by Sato et al. [26] is showing the stability of the results with respect to variations of these parameters around the reported values.

**Figure 2:**
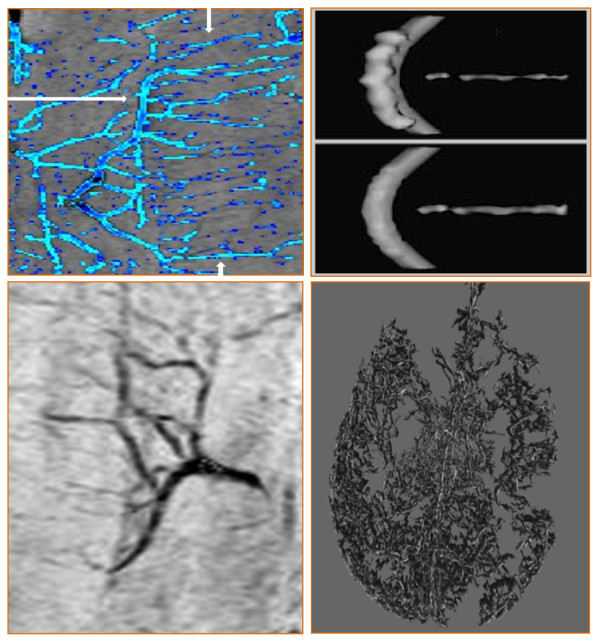
**Multi-scale filter approach segmentation steps are presented by: a) recovery of line structures of various widths, b) Hessian eigenvalues (i.e., linear intensity minima) removal of the effects of non-linear structures and recovery of the original structures as described by Sato et al., **[26]**c) removal of the effects of non-uniformity of contrast material, noise and artifacts, and d) 3-dimensionality**

Multi-scale filter approach segmentation steps are represented in Figure 2. To assess the performance of the vesselness filter with respect to signal to noise ratio (SNR), the filter output was thresholded and overlaid onto the original mIP data for visual inspection of the segmentation process. Visual comparison with mIP images confirmed that the line-filter had high detection sensitivity with respect to small vessels, vessel continuity, and the reduction of noise and artifacts, both pre- and post-contrast injection. Note that, in order to obtain optimal 3D projections of the datasets, the brain was extracted (i.e., the skull was removed) by using the FSL-brain extraction tool [27].

**Figure 3:**
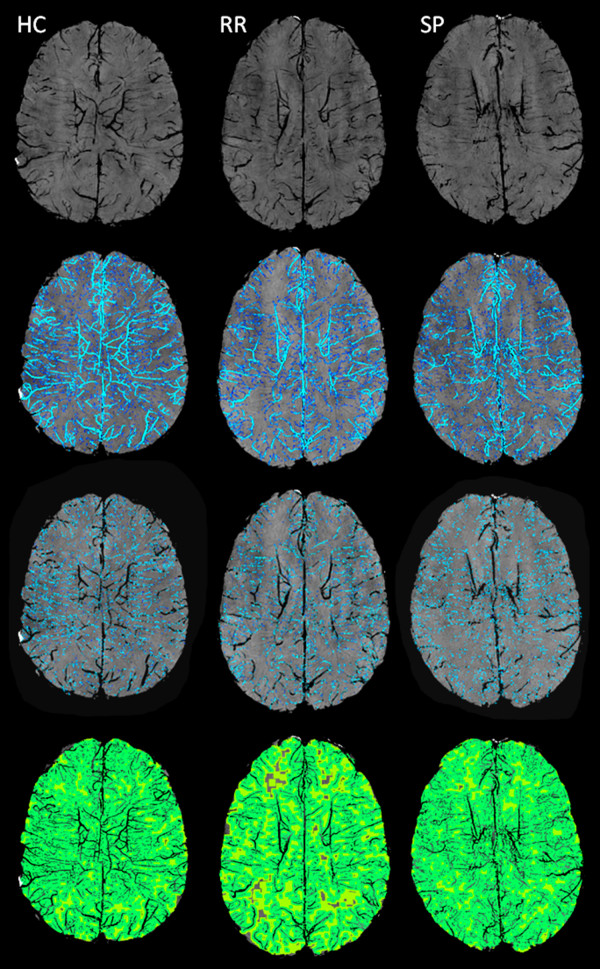
**The images display minimum projection (mIP) (horizontal upper row), apparent total venous volume (ATVV) (horizontal middle upper row), ATVV with a diameter < .3 mm (horizontal middle lower row) and average distance from voxel (DFV) map (horizontal lower row) for a healthy control (HC) (vertical left column), relapsing-remitting (RR) (vertical middle column) and secondary progressive (SP) (vertical right column) multiple sclerosis patients**. The images of ATVV and ATVV with a diameter < .3 mm display the differences between HC, RR and SP MS patients [please note decreased ATVV (represented in light blue for ATVV and light green for ATVV with a diameter < .3 mm)] especially in the ventricular, periventricular white matter, deep gray matter and cortical structures between HC and MS patients, but no difference between RR and SP MS patients. The DFV maps show the differences between HC, RR and SP MS patients (please note that the green color represents distance closer to the veins, whereas the yellow and orange colors represent longer distance to the veins). RR and SP MS show more yellow and orange color compared to HC.

Apparent total venous volume (ATVV) measurement for total vein vasculature was performed in milliliters (ml) and the relative venous intracranial fraction (VIF) was calculated by normalization with respect to the intracranial volume as calculated by the FSL-brain extraction tool, to correct for head size (Figure 3).

The size of individual veins was measured in mm, and 4 groups were created according to their mean diameters: < .3 mm, .3-.6 mm, .6-.9 mm and > .9 mm. Classification of the vessels in the four size classes was performed by iterative erosion and dilation of the vein extraction map slice by slice. As a first step, an upsampled map was generated, doubling the number of voxels in each direction in the axial plane (from 768 × 576 to 1536 × 1152); consequently, at each iteration of the erosion and dilation process the vessels size was reduced or increased, respectively, by a voxel unit. By eroding and dilating the vessel mask and taking the difference from the original vessel mask, the linear structures that were a size smaller than the original voxel size (< .3 mm) were left (Figure 3). Iterating this process up to 4 times produced classification of the linear structures in the diameter classes < .3 mm, .3-.6 mm, .6-.9 mm and > .9 mm. At every iteration, a size filter was applied in order to avoid discontinuities in every size-class map due to local inhomogeneities or higher noise level. As a mIP image is used for the underlying processing, the vein volume measurements, while correlating with the actual vein volume in the brain, tend to increase vein visibility in the brain over other structures. In particular, this approach enhances the visibility of smaller veins over larger ones, as the former are projected over multiple neighboring slices, while the latter tend to be projected on themselves.

Each vein extraction map was verified by an experienced neuroimager, and any misclassifications were manually corrected.

As a last step, starting from the vein extraction maps, voxel-wise minimum distance from vein (DFV) maps were calculated, defined as the distance of each voxel from the closest voxel classified as belonging to a vein; in such maps longer distance indicates lower local vein density (Figure 3). The average value of this map was calculated over the whole brain, providing an average-distance-from-vein score.

Interclass correlation coefficient (ICC) values for all vein extraction indices were calculated on a set of 5 HC and 5 MS patients that were scanned twice over a one week period, both on pre- and post-contrast SWI images.

### SWI pre- and post-contrast experiment

**Figure 4:**
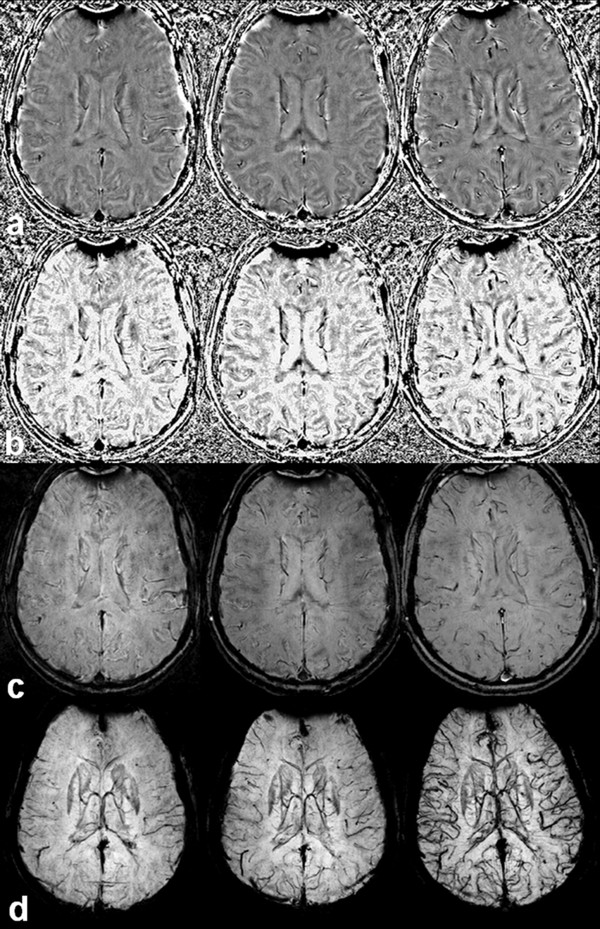
**Contrast enhanced susceptibility weighted imaging (SWI) increases detection of signal hypointensities on SWI venography in patient with multiple sclerosis, in T2 lesions and normal appearing white matter **[30]. Presented are pre-contrast (left), single dose post-contrast (middle) and triple dose post-contrast (right) images. The rows represent: a) high-pass filtered SWI, b) SWI phase mask, c) magnitude image multiplied 6 times by the phase mask, and d) minimum Intensity Projection image over 13 slices. Larger number and better defined signal hypointesities are detected with single and triple dose post-contrast SWI compared to pre-contrast SWI.

It has been reported that SWI venography can be further enhanced through the injection of a contrast agent, producing increased sensitivity and image quality [28,29]. The amount of accumulated phase, and therefore contrast, is proportional to the concentration of the contrast agent [25]. We previously reported in a pilot study that contrast-enhanced SWI significantly increases the number and volume of signal hypointensities on SWI venography in T2 lesions and in normal appearing WM (Figure 4) [30]. In order to investigate the SWI VVV parameter differences between pre- and post-contrast SWI additionally, 48 MS patients and 7 HC underwent both sequences in this study.

### Lesion and atrophy analyses

T2 hyperintense, T1 hypointense and gadolinium lesion volumes (LVs) were outlined using a semi-automated edge-detection contouring-thresholding technique [31].

Global and regional brain atrophy was determined with corrections for T1-hypointensity misclassification using an in-house developed in-painting program, on the 3D T1 weighted scans using SIENAX version 2.6., as described previously [32].

### Statistical analyses

Statistical analyses were performed using the Statistical Package for Social Sciences (SPSS, version 16.0). For descriptive statistics and estimates of CCSVI prevalence and its severity, t-tests, Fisher's exact tests and the Mann-Whitney rank sum U-test were used. Given that not all VVV indices were normally distributed, we employed non-parametric statistics. SWI venography differences between groups were assessed using the Mann-Whitney rank sum U-test. Differences between pre- and post-contrast SWI VVV measures in individual MS patients were assessed using the Wilcoxon signed rank test. Correlation analysis between severity of CCSVI (VHISS) and pre- and post-contrast VVV parameters was performed using the Spearman rank correlation coefficient. The primary null hypothesis was that there is no association between CCSVI and MRI brain parenchyma vein pathology. As a robust test of the hypothesis that does not assume a relationship between CCSVI and brain parenchyma vein pathology, we examined differences in MRI variables in the entire cohort of subjects with and without CCSVI. If significance was found, we performed the same comparison in individual diagnostic subgroups and MS disease subtypes. Due to multiple comparisons, only a nominal two-tailed p-value < .01 was considered statistically significant.

## Results

### Demographic and clinical characteristics

Table 1 shows demographic, clinical and conventional MRI characteristics of MS patients and HC. The median number of days between Doppler and MRI examination was 3. There were no age or sex differences between MS patients and HC. As expected, the SP patients were older, had longer disease duration and higher EDSS (p < .0001). There were no significant demographic and clinical differences between those who participated in the SWI venography portion of the CTEVD study and those who did not. There were no significant age, sex, disease duration or conventional MRI characteristics differences between the 48 MS patients and 7 HC who obtained pre- or post-contrast SWI scans and the entire study cohort. Of the 59 MS patients participating in the study, 56 were on disease-modifying therapy.

**Table 1:** Demographic and clinical characteristics in multiple sclerosis patients and healthy controls.

	MS (n = 59)	HC (n = 33)	p	RR (n = 41)	SP (n = 18)	p
Female gender, n (%)	43 (72.9)	20 (60.6)	NS	28 (68.2)	15 (83.3)	NS

Age in years, mean (SD)	44.6 (11)	41.3 (11.1)	NS	40.5 (9.6)	53.8 (8.1)	< .0001

Age at onset, mean (SD)	30.9 (9.8)	----	---	30.6 (8.6)	31.7 (12.3)	NS

Disease duration, mean (SD)	13.3 (10)	---	---	9.5 (5.7)	21.9 (12)	< .0001

Expanded Disability Status Scale, mean (SD) median	3.2 (1) 2.5	---	---	2.1 (0.9) 2.0	5.9 (1.1) 6.0	< .0001

T2 lesion volume, mean (SD)	12.8 (15.2)	2.3 (2.5)	< .0001	8.9 (7.7)	21.9 (23)	.0014

T1 lesion volume, mean (SD)	2.5 (6.5)	---	---	1.3 (2.8)	5.5 (10.6)	.016

Gadolinium lesion volume, mean (SD)	0.07 (0.4)	---	---	0.1 (0.5)	0.01 (0.04)	NS

Normalized brain volume, mean (SD)	1545.1 (107.3)	1642.4 (81.8)	< .0001	1575.2 (94.5)	1476.4 (105.3)	.002

Normalized gray matter volume, mean (SD)	828 (97.2)	882.4 (112.8)	.022	855.8 (93.5)	764.7 74.5)	< .0001

Normalized white matter volume, mean (SD)	717.1 (68.5)	725.6 (72.6)	.018	719.5 (73)	711.7 (58.3)	NS

MS - Multiple Sclerosis; HC - Healthy Controls; RR - Relapsing-Remitting; SP - Secondary Progressive; EDSS - Expanded Disability Status Scale; NS - non significant

All volumes are expressed in milliliters.

The differences between the study groups were tested using the chi-square, Student's t-test and the Mann-Whitney rank sum test.

### Scan-rescan dataset

ICC was slightly higher for the post-contrast SWI segmentation VVV measures than for the pre-contrast measures (Table 2). All VVV measures showed high ICC values and were significant (p < .003). The highest ICC were shown for the ATVV (.0.891) with vein sizes > .6 mm being most reproducible. The VIF and DFV ICC values ranged between 0.738 and 0.815.

**Table 2:** Scan-rescan reproducibility in 5 multiple sclerosis patients and 5 healthy controls who underwent pre- and post-contrast susceptibility-weighted imaging scan one week apart.

	Pre-contrast SWI ICC		Post-contrast SWI ICC	
ATVV for all vein sizes	0.891	< .001	0.912	< .001
ATVV with diameter < .3 mm	0.812	.002	0.825	.001
ATVV with diameter < .3-.6 mm	0.888	< .001	0.907	< .001
ATVV with diameter < .6-9 mm	0.923	< .001	0.941	< .001
ATVV with diameter > .9 mm	0.935	< .001	0.942	< .001

Venous intracranial fraction	0.791	.002	0.815	.001

Average distance from vein	0.738	.003	0.777	.002

ATVV - apparent total venous volume; mm - millimeters

Intraclass correlation coefficient and p values are presented.

### CCSVI status assessment

All subjects had the 5 VH criteria assessed. Table 3 shows the CCSVI classifications by disease group and by disease subtype. Prevalence of CCSVI was significantly higher in MS patients compared to HC (p < .0001). The measure of CCSVI severity (VHISS) was significantly higher in MS patients compared to HC (p < .0001).

**Table 3:** Presence and severity of CCSVI in multiple sclerosis patients and healthy controls.

	MS (n = 59)	HC (n = 33)	p	RR (n = 41)	SP (n = 18)	p
No CCSVI, n (%)	12 (21.3)	27 (81.8)	< .0001	11 (26.8)	1 (5.6)	NS
CCSVI, n (%)	47 (79.7)	6 (18.2)		30 (73.2)	17 (94.4)	

VHISS, mean (SD)	4.9 (3.1)	1.5 (1.6)	< .0001	5.2 (3.6)	4.2 (1.4)	NS

CCSVI - chronic cerebrospinal venous insufficiency; VHISS - venous hemodynamic insufficiency score; MS - Multiple Sclerosis; HC - Healthy Controls; RR - Relapsing-Remitting; SP - Secondary Progressive; NS - non significant

The differences between the study groups were tested using Fisher's exact tests and the Mann-Whitney rank sum test. All subjects had the 5 venous hemodynamic criteria correctly assessed.

There was a higher prevalence of CCSVI in SP compared to RRMS patients, but this difference did not reach significance.

### Vein vasculature visibility assessment

Table 4 shows quantitative assessment of brain parenchyma pre- and post-contrast VVV according to disease group and disease subtype. Patients with MS showed decreased ATVV, ATVV of veins with a diameter < .3 mm and increased DVF (all p < .0001). There was also a trend for lower VIF in MS patients (p = .049). Similar pre- and post-contrast VVV values were detected in 48 MS patients who underwent both pre- and post-contrast SWI. However, in 7 HC who underwent pre- and post-contrast SWI, significantly increased ATVV and ATVV of veins were found, as well as DFV on post-contrast scans. Increased ATVV occurred in veins with a diameter < .3 mm and a trend for increased ATVV of veins occurred in veins with a diameter < .3-.6 mm and .6-.9 mm.

**Table 4:** Quantitative brain parenchyma venous vascular assessment in multiple sclerosis patients and healthy controls on pre- and post-contrast susceptibility-weighted images.

	MS (n = 59) Pre-contrast Mean (SD)	MS (n = 48) Post-contrast Mean (SD)	HC (n = 33) Pre-contrast Mean (SD)	HC (n = 7) Post-contrast Mean (SD)	p-value^a^	p-value^b^	p-value^c^	p-value^d^
ATVV for all vein diameters	66.9 (16.6)	68.9 (15.1)	82.9 (17.4)	91.6 (18.1)	< .0001	< .0001	NS	.01
ATVV with diameter < .3 mm	45 (10)	48.9 (7.9)	53.8 (10.2)	61.3(7.7)	< .0001	< .0001	NS	.01
ATVV with diameter .3-.6 mm	15.5 (4.4)	16.3 (4.6)	17.4 (4.7)	21.2 (5.2)	.068	.046	NS	.018
ATVV with diameter .6-.9 mm	4.5 (1.8)	4.5 (2)	4.9 (2)	5.3 (2)	NS	NS	NS	.044
ATVV with diamater > .9 mm	1.9 (0.9)	1.9 (1.2)	2.1 (0.9)	2.3 (0.9)	NS	NS	NS	NS

Venous intracranial fraction	0.061 (0.02)	0.062 (0.01)	0.067 (0.01)	0.069 (0.01)	.049	.05	NS	NS

Average distance from vein	1.24 (0.2)	1.23 (0.4)	1 (0.1)	0.94 (0.1)	< .0001	< .0001	NS	.018

	**RR (n = 41)** **Pre-contrast** **Mean (SD)**	**RR (n = 32)** **Post-contrast** **Mean (SD)**	**SP (n = 18)** **Pre-contrast** **Mean (SD)**	**SP (n = 9)** **Post-contrast** **Mean (SD)**	**p-value^e^**	**p-value^f^**		

ATVV for all vein diameters	64.5 (18.7)	66.5 (17.2)	72.4 (8.3)	74.2 (6.2)	.06	NS		
ATVV with diameter < .3 mm	43.4 (11.1)	47.7 (8.5)	48.6 (7.1)	51.5 (5.9)	.046	NS		
ATVV with diameter .3-.6 mm	14.9 (5)	15.4 (5.2)	16.9 (2.1)	18.3 (2)	NS	.048		
ATVV with diameter .6-.9 mm	4.2 (2.1)	4.2 (2.2)	5.2 (0.6)	5.2 (0.4)	NS	NS		
ATVV with diamater > .9 mm	1.8 (1.1)	1.7 (1.4)	2.3 (0.5)	2.1 (0.4)	NS	NS		

Venous intracranial fraction	0.058 (0.02)	0.059 (0.01)	0.068 (0.008)	0.068 (0.008)	.025	.052		

Average distance from vein	1.26 (0.2)	1.29 (0.4)	1.22 (0.2)	1.23 (0.2)	NS	NS		

MS - Multiple Sclerosis; HC - Healthy Controls; RR - Relapsing-Remitting; SP - Secondary Progressive; NS - non significant; ATVV - apparent total venous volume

ATVV values are in milliliters and average distance from vein in millimeters.

p value^a ^- represents pre-contrast SWI VVV measure differences between MS patients and HC, p value^b ^- represents differences between MS patients post-contrast and HC pre-contrast SWI VVV measures, p value^c ^- represents pre- and post-contrast SWI VVV measure differences in MS patients, p value^d ^- represents pre- and post-contrast SWI VVV measure differences in HC controls, p value^e ^represents pre-contrast SWI VVV measure differences between RR and SP patients, p value^f ^- represents post-contrast SWI VVV measure differences between RR and SP patients.

The differences between the study groups were tested using the Mann-Whitney rank sum test. The differences between pre-contrast and post-contrast SWI VVV measures in the same individuals were tested using the Wilcoxon signed rank test.

RRMS patients showed a trend for decreased pre-contrast ATVV, ATVV of veins with a diameter < .3 mm and VIF compared to SPMS (all p < .06). Similar post-contrast VVV values were found in 32 RR and 14 SP MS patients who underwent both pre- and post-contrast SWI (Table 4).

### Presence of CCSVI and vein vasculature visibility assessment

Table 5 shows quantitative brain parenchyma VVV in MS patients and HC, according to CCSVI status on pre-contrast SWI. Subjects diagnosed with CCSVI had significantly increased DFV (p < .0001), decreased ATVV and ATVV of veins with a diameter < .3 mm (p < .003) compared to subjects without CCSVI. There was also a trend for lower VIF (p = .044).

**Table 5:** Quantitative brain parenchyma venous vascular assessment in multiple sclerosis patients and healthy controls, according to CCSVI status on pre-contrast susceptibility-weighted images.

	Total CCSVI (n = 53) Mean (SD)	Total No CCSVI (n = 39) Mean (SD)	p-value
ATVV for all vein diameters (ml)	67.2 (17.8)	78.3 (16.6)	.003

ATVV with diameter < 0.3 mm (ml)	45.1 (11)	51 (9.4)	.003

Venous intracranial fraction	0.059 (0.02)	0.067 (0.01)	.044

Average distance from vein (mm)	1.25 (0.2)	1.1 (0.1)	< .0001

	**MS CCSVI** **(n = 47)**	**MS No CCSVI (n = 12)**	

ATVV for all vein diameters (ml)	65.1 (17.1)	70 (8)	NS

ATVV with diameter < 0.3 mm (ml)	44 (10.8)	47 (4.5)	NS

Venous intracranial fraction	0.059 (0.02)	0.066 (0.01)	.084

Average distance from vein (mm)	1.29 (0.2)	1.08 (0.02)	.01

CCSVI - chronic cerebrospinal venous insufficiency; MS - Multiple Sclerosis; NS - non significant; ATVV - apparent total venous volume; ml - milliliters; mm - millimeters

The differences between the study groups were tested using the Mann-Whitney rank sum test.

When only MS patients were investigated, significantly increased DFV (p = .01) and a trend for decreased VIF (p < .084) were found in subjects with CCSVI compared to subjects without CCSVI on pre-contrast SWI. These differences were more pronounced in RRMS subjects, who showed significantly increased DFV (p = .009) and a trend for lower VIF (p = .07). Similar post-contrast VVV differences between MS patients with and without CCSVI were found in 48 MS patients. Significantly increased DFV (1.28 vs. 1, p = .01) and a trend for decreased VIF (.061 vs. .066, p = .09) were found in MS patients with CCSVI compared to subjects without CCSVI.

No significant differences were observed between subjects with and without CCSVI in HC.

No significant differences were observed between subjects with and without CCSVI for other MRI measures.

### Severity of CCSVI and vein vasculature visibility and other MRI measurement assessments

**Figure 5:**
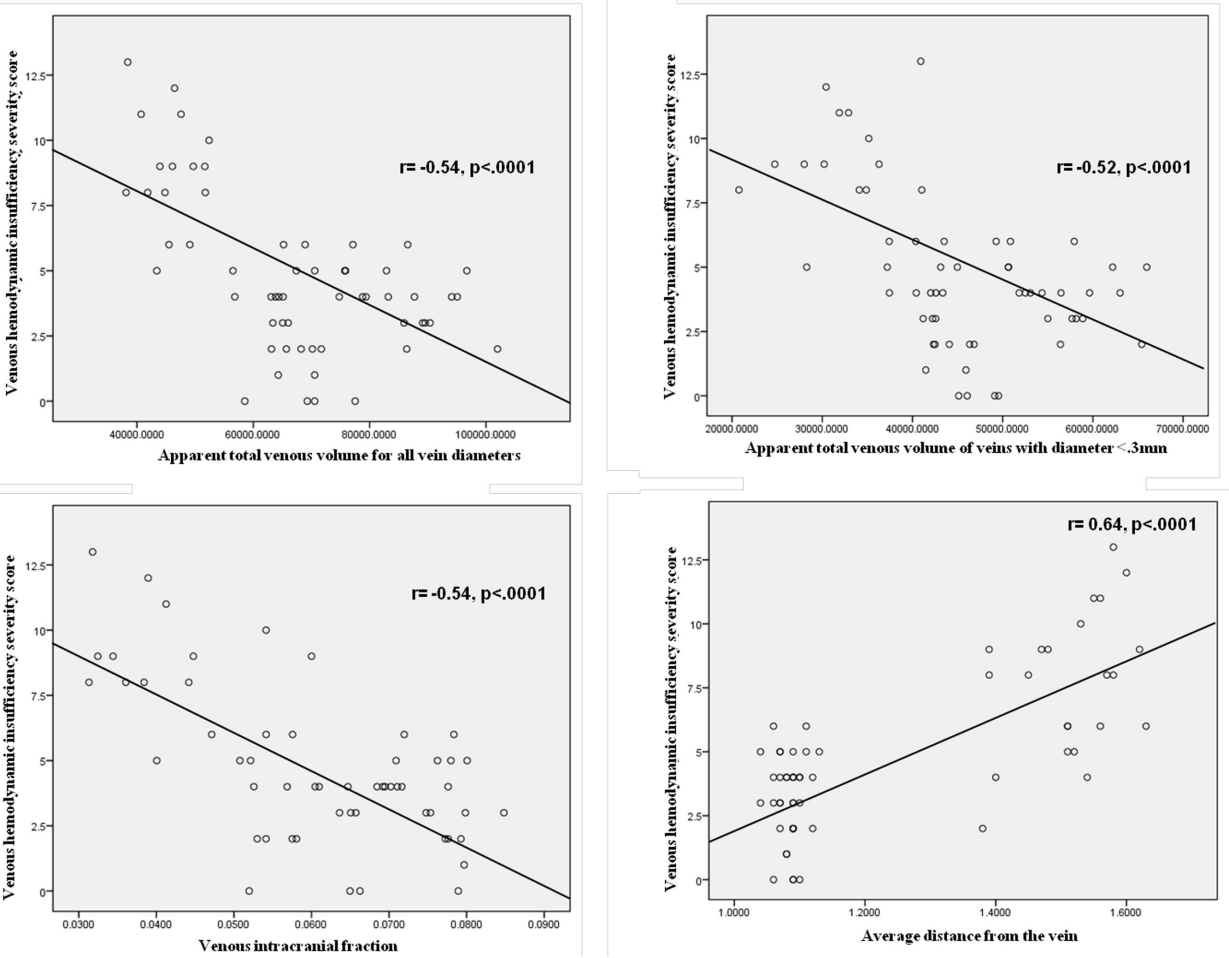
**Scatter plots of the pre-contrast SWI venography measures and severity of the CCSVI (VHISS) in multiple sclerosis patients**.

Table 6 and Figure 5 show the association between severity of CCSVI and quantitative assessment of brain parenchyma VVV on pre- and post-contrast SWI. In MS patients as a group, all quantitative brain parenchyma venous vascular indices, reflecting increased damage of venous vasculature, were significantly related to higher VHISS (p < .0001) on both pre- and post-contrast images. The association was particularly strong in RR, but not significant in SP MS patients alone.

**Table 6:** Relationship between the venous hemodynamic severity score (VHISS) and quantitative brain parenchyma venous vasculature in multiple sclerosis patients on pre-and post-contrast susceptibility-weighted images.

Pre-contrast	MS (n = 59)		RR (n = 41)		SP (n = 18)	
	r	p	r	p	r	p
ATVV for all vein diameters (ml)	-0.54	< .0001	-0.7	< .0001	-0.17	.500

ATVV with diameter < 0.3 mm (ml)	-0.52	< .0001	-0.67	< .0001	-0.24	.340

Venous intracranial fraction	-0.54	< .0001	-0.67	< .0001	-0.12	.653

Average distance from vein (mm)	0.64	< .0001	0.73	< .0001	0.21	.413

**Post-contrast**	**MS** **(n = 48)**		**RR** **(n = 36)**		**SP** **(n = 12)**	
	r	p	r	p	r	p

ATVV for all vein diameters (ml)	-0.46	< .0001	-0.47	< .0001	-0.23	.425

ATVV with diameter < 0.3 mm (ml)	-0.48	< .0001	-0.53	< .0001	-0.14	.722

Venous intracranial fraction	-0.57	< .0001	-0.6	< .0001	-0.28	.354

Average distance from vein (mm)	0.62	< .0001	0.72	< .0001	0.26	.324

MS - Multiple Sclerosis; RR - Relapsing-Remitting; SP - Secondary Progressive; ATVV - apparent total venous volume;

The relationship between VHISS and quantitative brain parenchyma venous was explored using the Spearman rank correlation coefficient.

No significant relationship was observed in HC between VHISS and VVV indices.

## Discussion

This is the first study to investigate the association between the presence and severity of CCSVI and venous vasculature in the brain parenchyma on SWI venography in patients with MS and in HC. There are three main findings in this study: 1) first, by examining only brain parenchyma VVV, we found that MS patients are compromised compared to HC. The novelty of our SWI approach using pre- and post-contrast SWI venography experiment is that its suggests that the reduced venous visibility in brain parenchyma of MS patients is not only the result of reduced metabolism, but may be affected by possible morphological changes in venous vasculature. This was not shown previously by using post-contrast SWI venography and only one previous study [10] showed similar qualitative (but not quantitative) pre-contrast SWI venography findings; 2) second, by quantitatively associating VVV with the presence or absence of CCSVI, we found significantly increased pre-contrast DFV, decreased ATVV and ATVV of veins with a diameter < .3 mm, and a trend for decreased VIF in subjects presenting with CCSVI; 3) third, MS patients with higher number of venous stenoses, indicative of CCSVI severity, showed significantly decreased VVV in the brain parenchyma on both pre- and post-contrast SWI. The pathogenesis of these findings has to be further investigated, but they suggest that decreased venous vasculature in brain parenchyma of MS patients is strongly related to presence and severity of CCSVI. These findings are important for better understanding of the MS pathogenesis, and the current findings support results of two previous pilot hemodynamic imaging studies (using PWI and CSF flow measurement) that showed that drainage problems in patients with MS are associated with presence and severity of CCSVI [18,19]. By quantitatively measuring the most important drainage pathway from brain parenchyma to the periphery, which consists of the veins themselves, we are confirming and extending the validity of these previous preliminary findings.

When MS patients' subtypes were compared, the association between the severity of CCSVI and altered pre- and post-contrast SWI VVV was stronger in RR than in SPMS patients, and RRMS patients also presented with a trend for more altered pre- and post-contrast SWI VVV compared to the SP group. The significance of these findings is unclear and should be further explored; however, it could indicate that VVV changes may occur very early on in the MS disease process.

This study did not explore the relationship between SWI-VVV and markers of inflammation and demyelination in patients with MS from the earliest clinical stages. It could be that the reduced venous visibility in brain parenchyma of MS patients, and consequently decreased venous vasculature, is secondary to the presence and severity of inflammatory and demyelinating lesions that are accumulating over time in MS patients. Further studies using conventional and non-conventional MRI measures are needed to determine how presence and severity of intrinsic lesion damage relates to changes in venous vasculature. Therefore, the pathogenetic role of our VVV findings should be interpreted with caution.

The automated vein extraction segmentation approach we have developed greatly diminishes the impact of individual operators. The algorithm was based on the vesselness filter described by Sato et al., [26], as this was proven by the authors to be stable and highly reproducible. The intrinsic advantage of this approach lies in its multi-dimensionality, allowing for the segmentation of vessels of different sizes, the ability to recover continuity of the structures despite higher local noise levels, and most of all the fact that it is not based on absolute signal intensity (zero order information) but rather on intensity gradients surrounding each voxel (second order information), giving sensitivity to the shape of the structures. The vesselness filter is integrated into the ITK library (Insight Segmentation and Registration Toolkit, http://www.itk.org/) and has been applied to vessel segmentation on various imaging techniques, including MRI, computerized tomography and optical computerized tomography.

The scan-rescan experiment data showed that SWI venography VVV indices we measured are highly reproducible (Table 2). As expected, post-contrast SWI VVV measures showed slightly better reproducibility results than pre-contrast measures. Of all different vein diameter ATVV measurements we performed, the veins with larger diameter (> .3 mm) showed better reproducibility than those < .3 mm. This is expected, as the segmentation outcome of veins < .3 mm will be most influenced by image quality, movement-related partial volume effects and image processing steps. These may be related to different parameters, including image contrast-to-noise ratio, unwrapping of phase images, filter characteristics (tuning parameters, threshold) of the Hessian filter, motion artifacts, and change in SNR. However, we inspected all segmentation output of the images visually. In addition, our reproducibility analysis did not show that the potential change of these imaging parameters affected the reproducibility of results over one week. On the other hand, VIF and DFV displayed a reduced reproducibility. For the VIF, the reason most likely lies in the intrinsic uncertainty in the estimation of the intra-cranial volume, which is used to normalize the VVV across subjects. For the DFV instead, reduced reproducibility is the effect of the enhanced vein visibility which characterizes the mIP maps.

SWI is very sensitive in detecting signals from substances with magnetic susceptibilities that are different from that of their neighbors. Consequently, SWI is able to detect tissue iron in the form of ferritin, hemosiderin and deoxyhemoglobin, [25,33,34] and is sensitive to the visualization of small veins in the brain [10]. SWI venography allows detailed visualization of cerebral veins in the brain parenchyma without the use of an exogenous contrast agent [9]. This is possible by exploiting the difference in magnetic susceptibility properties between oxygenated and deoxygenated hemoglobin. The abundance of the paramagnetic deoxyhemoglobin molecule in the venous blood results in increased local magnetic field inhomogeneity, which in turn leads to spin dephasing and signal loss on SWI venography, resulting in decreased VVV [10]. One of the key aims of this study was to address whether the SWI venography VVV differences between MS patients and HC are only a result of decreased oxygen utilization in MS patients (with correspondent decreased levels of venous deoxyhemoglobin), as previously proposed, [10] or if there were also morphological changes in the small veins that became atrophic and disappeared due to the MS disease process, possibly leading to permanent decrease of signal on SWI. Contrast-enhanced SWI significantly increases the visualization of number and volume of signal hypointensities on SWI venography [28,29] in T2 lesions and in normal appearing WM (Figure 4), [30] and may be an additional means of investigating whether SWI venography differences between MS patients and HC are due only to hypometabolic status or whether morphological changes of veins may be taking place in MS patients. 81.4% of the MS patients and 21.2% of HC included in this study underwent both pre- and post-contrast SWI sequence in order to further elucidate this important question. We demonstrated a very similar decrease in brain parenchyma VVV on pre- and post-contrast SWI parameters we examined in MS patients, but significantly increased ATVV and ATVV of veins with a diameter < .3 mm in HC was found, as expected (Table 4). The reduction of vascular visibility on pre-contrast SWI between MS patients and HC was previously observed and attributed to hypometabolic status in brain parenchyma of MS patients [10]. However, the pre- and post-contrast SWI venography experiment performed in the present study further extends understanding of this phenomenon and suggests that the reduced VVV in MS may be a combination of two main effects - reduced metabolism and morphological changes of the venous vasculature. Further cross-sectional and longitudinal studies are needed to better elucidate this phenomenon. Subtraction technique approaches may be useful for detecting whether the loss of signal occurs in the same regions between pre- and post-contrast SWI venography. In addition, region-specific analysis may shed light regarding key areas that are involved. Visual analysis performed in this study suggests that cortical and deep WM/GM regions may be the most affected (Figure 3).

A hypoxia-like condition has been evidenced in patients with MS [35,36]. It has been shown that hypoperfusion of the brain parenchyma in MS patients may precede disease onset [37,38]. Abnormal perfusion patterns within normal appearing WM and GM were demonstrated in MS patients [38-41]. Chronic inflammatory events related to local blood congestion and secondary hyperemia of the brain parenchyma are proposed as a cause of these hemodynamic abnormalities detected on perfusion MRI in patients with MS [39,42,43]. Whether reduced perfusion of the WM and GM in MS patients is a sign of vascular pathology, decreased metabolic demand [36] or precipitated hemodynamic changes in the extra-cranial venous pathways [17] is not clear at this time. We recently reported in a pilot study a significant relationship between the severity of CCSVI and hypoperfusion in the brain parenchyma of 16 MS patients, but not in 8 healthy controls [17].

One of the key findings in this study is that MS patients presenting with CCSVI (and with increased severity of CCSVI, as measured by VHISS) showed decreased pre- and post-contrast VVV in brain parenchyma on SWI venography. These findings confirm results of earlier studies, [38-42] all of which observed significantly lower perfusion in the brain parenchyma of MS patients compared with controls. It could be hypothesized that decreased venous outflow from the brain parenchyma to the periphery would lead to increased intracranial pressure and subsequent venous stasis, especially of the small vein vasculature. Venous pressure was not measured in the current study; however, a recent study showed no increased intracranial venous pressure in MS patients [44]. Nevertheless, increased venous pressure in the subarachnoid space of MS patients cannot be excluded at this time. Several authors have hypothesized that reduced venous drainage outflow in MS may increase intra-capillary oncotic pressure, which would lead to decreased capillary permeability toward the extra cellular compartment and consequent intra-tissue accumulation of toxic metabolites [12,45]. In the present study, the association between presence and severity of CCSVI was particularly strong with VVV indices of small veins. We showed that there was a trend for differences between patients and controls for veins with a diameter < .6 mm. Hypoxia arising from stasis in the veins might therefore induce morphological changes which could result in occlusion and atrophy of these veins. Therefore, the most plausible explanation of our findings would be that reduced outflow from small vessels to the periphery--independent of whether it is primary or secondary to CCSVI or inflammatory and demyelinating lesions--leads to destruction of smaller veins and consequent loss of signal visibility on SWI venography. The pathogenesis of this process remains unanswered at this time and should be explored together with dynamic of regional lesion accumulation over time.

This loss of vasculature suggests that rerouting of the intracranial venous blood flow is probably taking place. If CCSVI is secondary to various vascular, infective and inflammatory processes (this hypothesis could explain the presence of CCSVI in HC), then the tendency to be chronic in its development may help explain the temporal dissociation between the loss of the VVV and no development of intracranial hypertension. In that context, hemodynamic compensatory mechanisms may play a key role. One such mechanism could relate to the development of extra-cranial collateral circulation [14,16] or altered CSF dynamics [17] that would compensate for altered primary outflow pathways.

Some recent reports have presented evidence against the CCSVI hypothesis in MS using Doppler and MRV assessments [46-51]. The conflicting reports of CCSVI-related venous abnormality prevalence findings between different studies using non-invasive and invasive imaging techniques emphasize the urgent need for better understanding of these anomalies. It is also important to place CCSVI in the context of other known associations in MS, with the most well established MS risk factors and clinical and MRI outcomes. The current study provides important evidence that extra-cranial venous abnormalities may be related to intra-cranial brain parenchyma reduced venous visibility. The dynamic of these findings should be further explored.

There are a number of limitations to the present study. One relates to the use of the proposed CCSVI criteria, [14] which could be insufficient to adequately describe the cerebral venous outflow due to the lack of assessment of functional data on blood flow velocity and blood volume flow [46]. Evaluation of blood flow velocity and blood volume flow may offer more complete status of the cerebral venous outflow in relation to decreased brain parenchyma SWI VVV in patients with MS. Another limit of the study is related to its cross-sectional design and to the relatively small number of subjects studied, especially of HC and MS patients at the early stage of the disease. Due to the nature of SWI phase imaging and associated "blooming" effects, our measurements of venous volume are inherently relative rather than absolute. Finally, the validation of our VVV method should be performed against either simulations or phantom measurements.

## Conclusion

In conclusion, the current study provides an initial step in better understanding of how decreased venous vasculature in brain parenchyma of MS patients may be related to the pathogenesis of MS. Further studies need to confirm and elucidate our findings.

## Competing interests

The authors declare that they have no competing interests regarding study in question. Robert Zivadinov received personal compensation from Teva Neuroscience, Biogen Idec, EMD Serono and Questcor Pharmaceuticals for speaking and consultant fees. Dr. Zivadinov received financial support for research activities from Biogen Idec, Teva Neuroscience, Genzyme, Bracco, Questcor Pharmaceuticals and EMD Serono. Bianca Weinstock-Guttman received personal compensation for consulting, speaking and serving on a scientific advisory board for Biogen& Idec, Teva Neuroscience and EMD Serono. Dr. Weinstock-Guttman also received financial support for research activities from NMSS, NIH, ITN, Teva Neuroscience, Biogen Idec, EMD Serono, and Aspreva. David Hojnacki has received speaker honoraria and consultant fees from Biogen Idec, Teva Pharmaceutical Industries Ltd., EMD Serono, Pfizer Inc, and Novartis. Guy U. Poloni, Karen Marr, Claudiu V. Schirda, Christopher R. Magnano, Ellen Carl, Niels Bergsland, Cheryl Kennedy, Clive B. Beggs and Michael G. Dwyer have nothing to disclose.

## Authors' contributions

RZ, GUP, KM, CVS, CRM, EC, NB, DH, CK, CBB, MGD, BWG have made substantial contributions to conception and design, or acquisition of data, or analysis and interpretation of data. RZ, GUP, CBB, MGD, BWG have been involved in drafting the manuscript, while KM, CVS, CRM, EC, NB, DH, CK revised it critically for important intellectual content. All authors have given final approval of the version to be published.

## Pre-publication history

The pre-publication history for this paper can be accessed here:

http://www.biomedcentral.com/1471-2377/11/128/prepub
